# Supplementary material for: Parabrachial tachykinin1-expressing neurons involved in state-dependent breathing control
Source: Nat Commun. 2023 Feb 21;14:963. doi: 10.1038/s41467-023-36603-z (PMC9944916; doi:10.1038/s41467-023-36603-z)
Supplement: Supplementary file 1 — Supplementary Information [file 41467_2023_36603_MOESM1_ESM.pdf]

Parabrachial tachykinin1-expressing neurons involved in state-dependent breathing control

Joseph W. Arthurs[1,2], Anna J. Bowen[1], Richard D. Palmiter[1], Baertsch NA[2,3]*

[1]Howard Hughes Medical Institute and Department of Biochemistry, University of Washington, Seattle WA 98195
[2]Center for Integrative Brain Research, Seattle Children's Research Institute, Seattle WA 98101
[3]Pulmonary Critical Care and Sleep Medicine, Department of Pediatrics, University of Washington, Seattle WA

Supplementary Figures

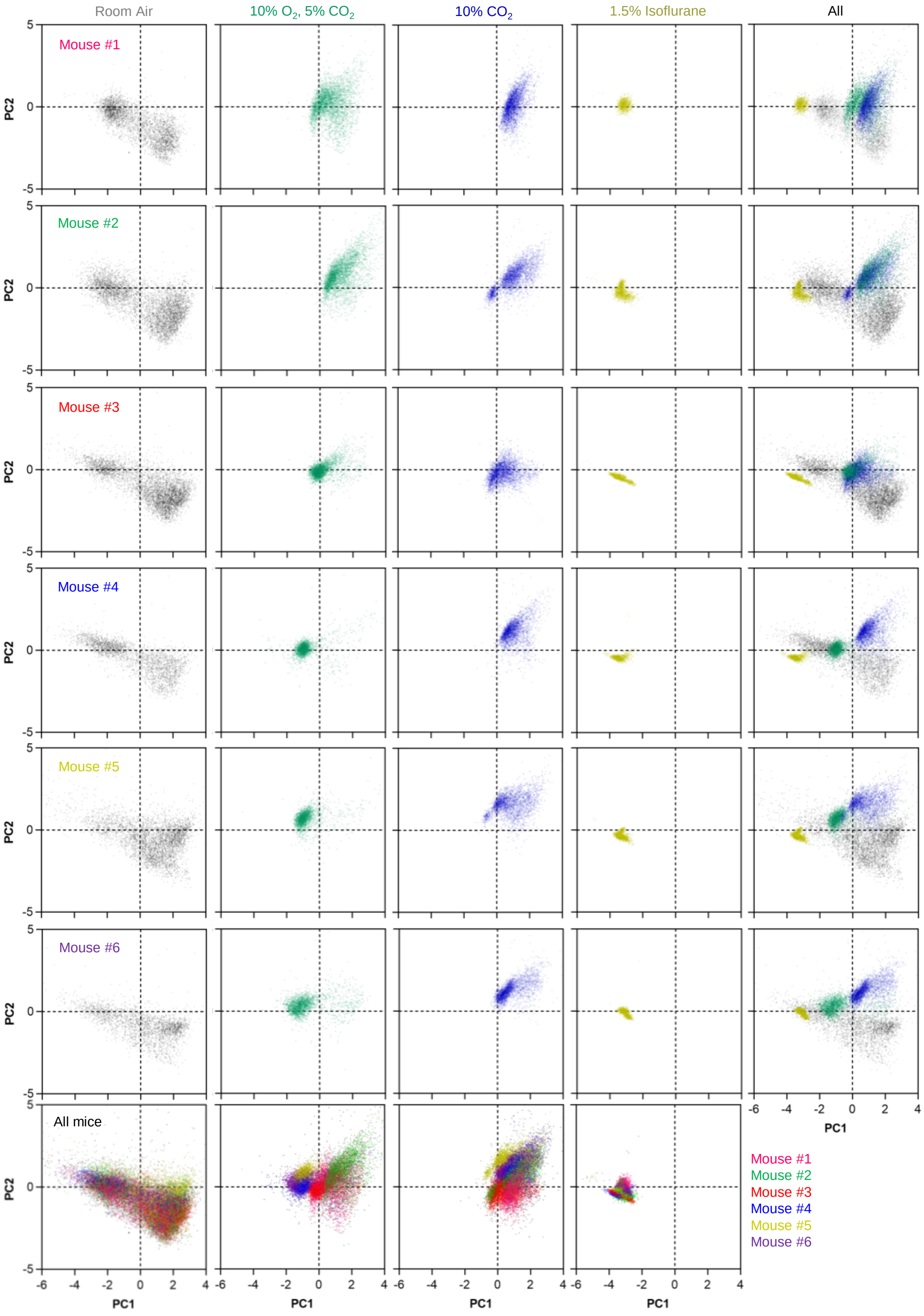

Figure S1: Rapid dynamic breathing patterns characterize the awake, normally behaving, state. PCA showing breathing patterns for each mouse when awake under control conditions (room air; gray, when physiological demands are increased and chemoreflexes are activated with 5% CO_2, 10% O_2 (green) or 10% CO_2, 21% O_2 (blue), or when anesthesia is induced with 1.5% isoflurane in room air (gold). Far right panels show overlay of PC scores of breaths form each condition for each individual mouse. Bottom panels show combined breaths in each condition color coded by replicate.

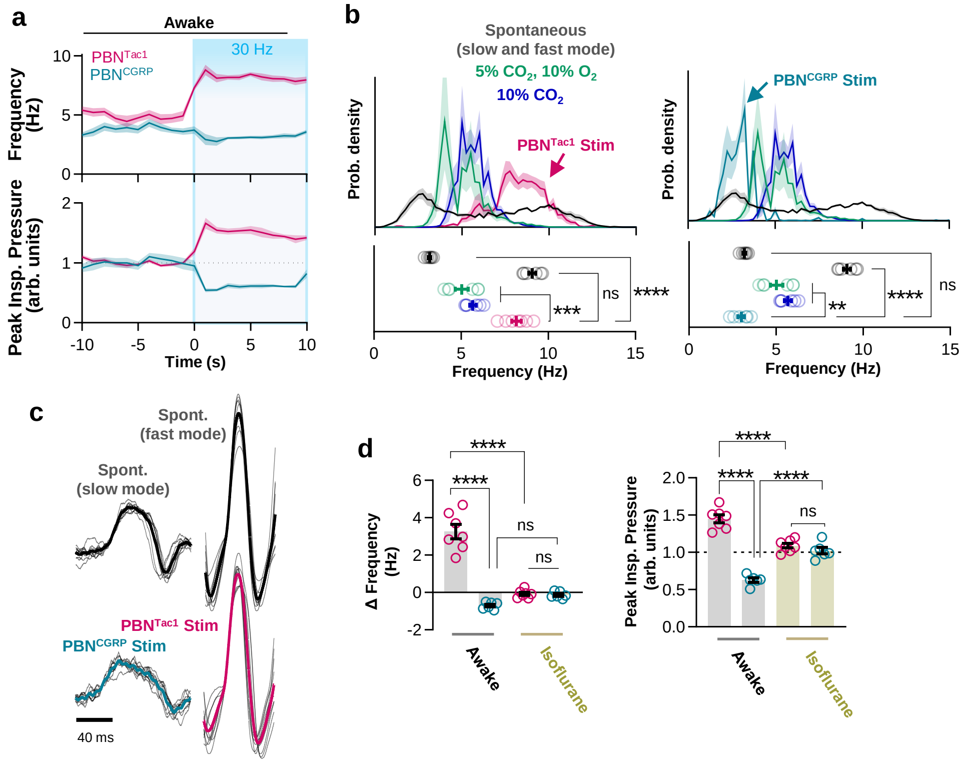

Figure S2: Peptidergic PBN subpopulations have opposing effects on breathing. **a**) Comparison of breathing frequency (top) and peak inspiratory pressure (bottom; normalized to light OFF condition) during 30-Hz photostimulation of Tac1 (n=7) or CGRP (n=6) PBN neurons. **b**) Probability density histograms and mean values from each mouse comparing breathing frequencies under spontaneous conditions (slow and fast mode) or during near maximal chemoreflex drive (5% CO_2, 10% O_2 or 10% CO_2; n=6 for each condition) with frequencies evoked during stimulation of Tac1 (top; n=7 mice) or CGRP (bottom; n=6 mice) neurons in the PBN. One-way ANOVA with Dunnett's T3 multiple comparisons tests. **c**) Overlayed breath waveforms with average (bolded line) comparing slow and fast mode breaths generated in the awake state under control conditions with breath waveforms generated during 30-Hz photoactivation of CGRP or Tac1 neurons in the PBN. **d**) Average changes in frequency and peak inspiratory pressure evoked by activation of Tac1 (n=7) or CGRP (n=6) PBN neurons in the awake or anesthetized state. Two-way RM ANOVA with Bonferroni's multiple comparisons tests. * p<0.05; ** p<0.01; *** p<0.001; **** p<0.0001. Means±SE. Source data and statistical details provided in Source Data file.

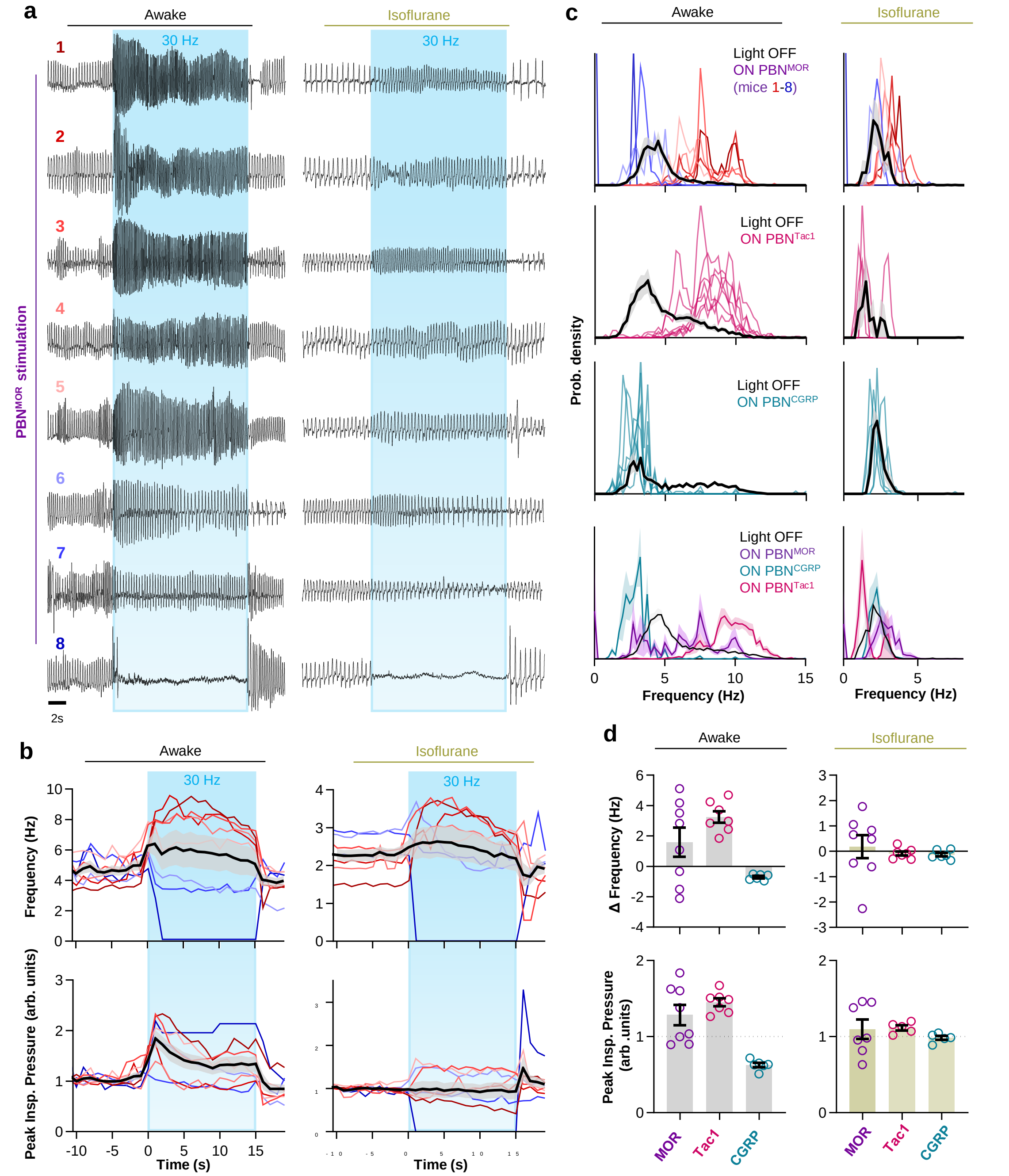

Figure S3: Respiratory effects of MOR PBN neurons. a) Plethysomgraph recordings from n=8 *Oprm1*$^{Cre/+}$ mice during bilateral 30-Hz stimulation of MOR expressing neurons in the PBN while awake (left) and in the same mice following induction of isoflurane anesthesia (right). **b**) Quantified breathing frequency (top) and peak inspiratory pressure (bottom) showing individual responses (red-blue) and means±SE (black) to photostimulation of MOR PBN neurons in the awake or anesthetized state. **c**) Probability density histograms showing individual responses to stimulation of MOR (red-blue), Tac1 (magenta), and CGRP (cyan) neurons versus the light OFF condition (means±SE; black) while awake (left) or anesthetized (right). Average data for all groups is compared in the bottom histogram (Light OFF conditions pooled). **d**) Average changes in breathing frequency and peak inspiratory pressure during photostimulation of MOR (n=8), Tac1 (n=7), and CGRP (n=6) PBN neurons while awake (left) and under isoflurane anesthesia (n=8, MOR; n=5, Tac1; n=5, CGRP). Means±SE. Source data and statistical details provided in Source Data file.

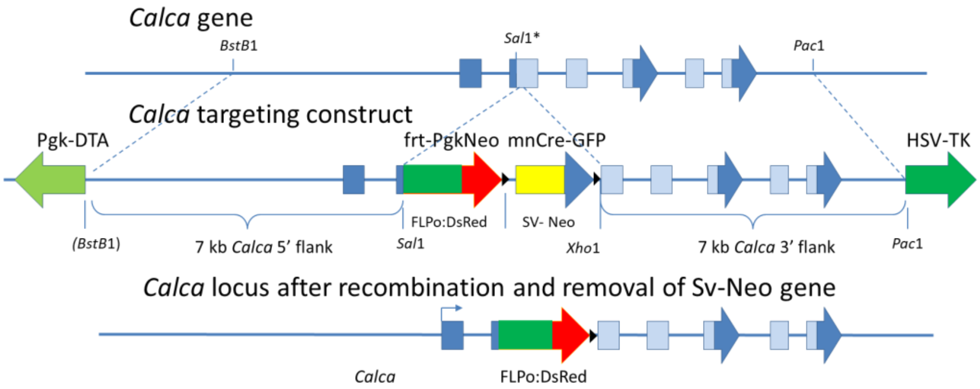

Figure S4: Generation of Calca$^{FLPo:DsRed}$ mouse line. A *Sal*l site was introduced at the start codon in exon 2 of the *Calca* gene in a 14-kb subclone from a C57BL/6 BAC clone. It was engineered to have Pgk-DTA and HSV-TK gene at its flanks (for negative selection). A loxP-flanked SV-Neo was inserted just 3' of the *Sal*1 site and a FLPo:DsRed cassette was inserted into the Sal1 site to create a targeted construct that was linearized with Asc1 and electroporated into G4 (C57BL/6 x SJL hybrid) ES cells. ES cells with correct targeting were identified, positive clones were injected into blastocysts, and transferred to pseudo-pregnant females. Mice from one clone that gave germ-line transmission were bred to *Meox*^{Cre} mice to delete the SV-Neo gene.

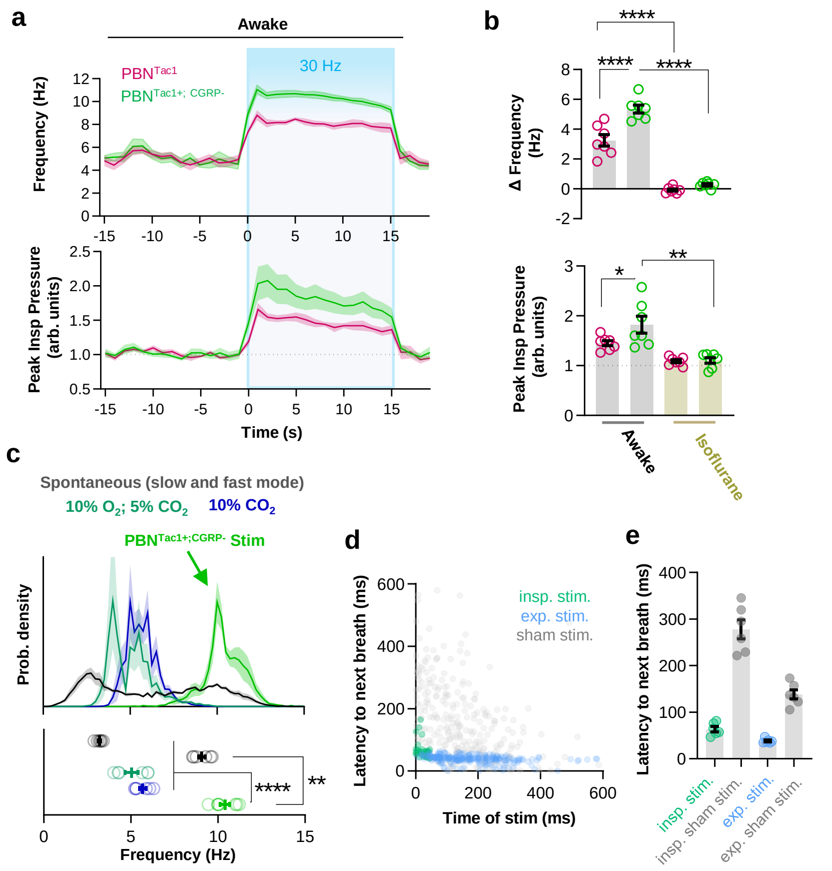

Figure S5: Respiratory effects of Tac1 PBN neurons are enhanced when CGRP PBN neurons are excluded. a) Comparison of breathing frequency (top) and peak inspiratory pressure (bottom; normalized to light OFF condition) during 30-Hz photostimulation of all Tac1 PBN neurons (n=7 mice) vs only Tac1 neurons that do not co-express CGRP (Tac1+; CGRP-) (n=7 mice). **b**) Average changes in frequency and peak inspiratory pressure evoked by activation of all Tac1 (n=7 mice) or Tac1+; CGRP- (n=7 mice) PBN neurons. Two-way RM ANOVA with Bonferroni's post-hoc multiple comparisons tests. **c**) Probability density histograms (top) and mean values from each mouse (bottom) comparing breathing frequencies under spontaneous conditions (slow and fast mode) or during near maximal chemoreflex drive (5% CO_2, 10% O_2 or 10% CO_2; n=6 for each condition) with frequencies evoked during stimulation of Tac1+; CGRP- neurons in the PBN (n=7 mice). One-way RM ANOVA with Tukey's multiple comparisons tests. **d**) Latencies from the onset of a brief 25-ms photostimulation of Tac1+; CGRP- PBN neurons to the onset of the subsequent breath vs. the time of the stimulation relative to the onset of the preceding breath (n=6 mice). **e**) Average latencies from 25-ms stimulations (or sham stimulations; gray) occurring during inspiration (green) or expiration (blue) to the subsequent breath for each mouse (n=6 mice). * p<0.05; ** p<0.01; *** p<0.001; **** p<0.0001. Means±SE. Source data and statistical details provided in Source Data file.

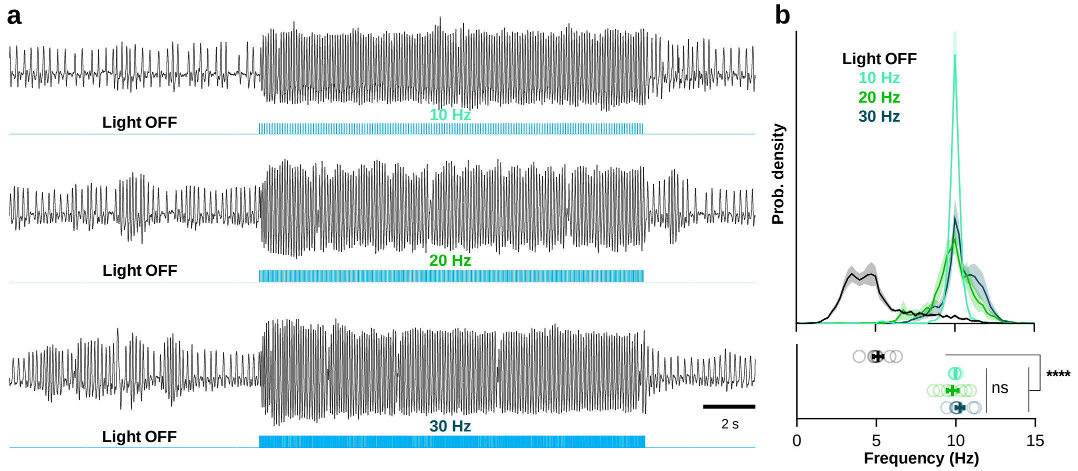

Figure S6: Breathing responses to increasing stimulation frequencies of Tac1+; CGRP- PBN neurons. a) Representative plethysmography recordings from an awake freely-behaving mouse during 10-, 20-, and 30-Hz stimulation of Tac1+; CGRP- PBN neurons. **b**) Quantified changes in breathing frequency comparing light OFF conditions to 10-, 20-, and 30-Hz stimulation (n=7). One-way RM ANOVA with Tukey's multiple comparisons tests. ns = not significant; * $p<0.05$; ** $p<0.01$; *** $p<0.001$; **** $p<0.0001$. Means±SE. Source data and statistical details provided in Source Data file.

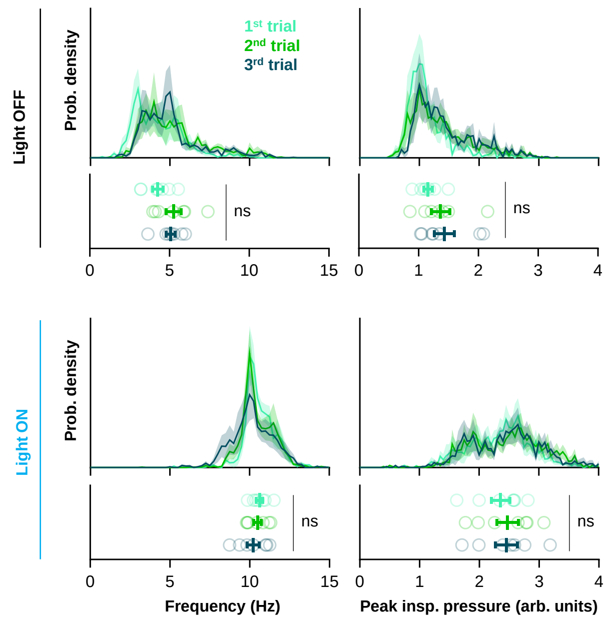

Figure S7: Breathing responses during repeated trails of Tac1+; CGRP- PBN neuron stimulation. Breathing frequency (left) and peak inspiratory pressure (right) during Light OFF (top) and Light ON (bottom) conditions over three consecutive 15-s trials of 30-Hz Tac1+; CGRP- stimulation (n=7). No significant differences in breathing patterns over repeated trials were observed. One-way RM ANOVA with Tukey's multiple comparisons tests. ns = not significant. Means±SE. Source data and statistical details provided in Source Data file.

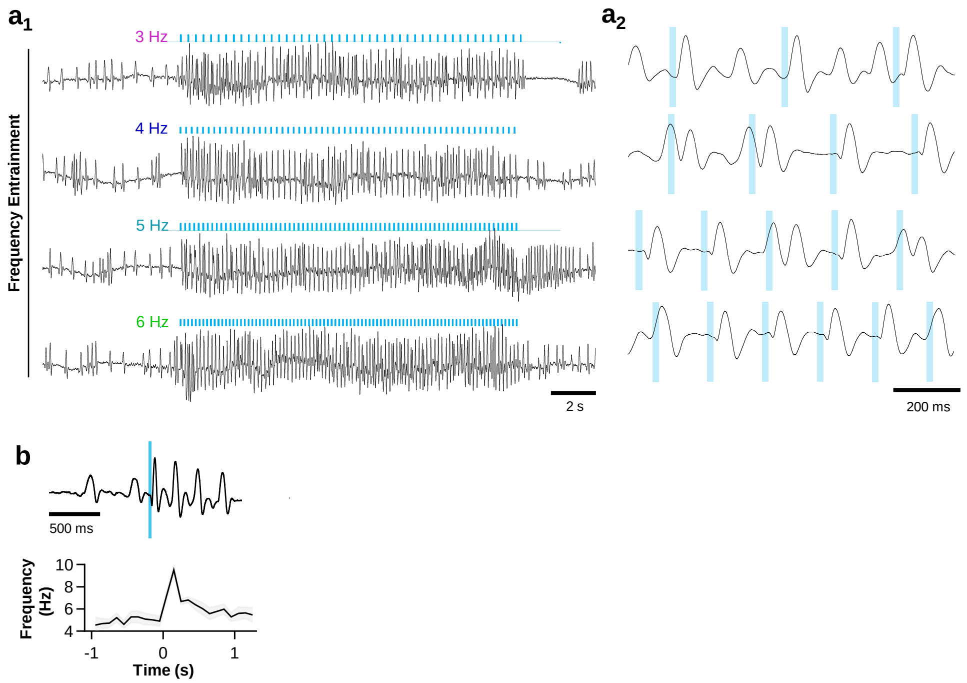

Figure S8: Changes in breathing pattern during stimulation of Tac1+; CGRP- PBN neurons at frequencies <7 Hz. $a_{1\text{-}2}$) Example plethysmograph recordings during 3-, 4-, 5-, and 6-Hz stimulations in an awake freely-behaving mouse. Note the poor entrainment due to multiple breaths/stimulation at lower frequencies. **b**) Example plethysmograph recording during a single brief (25 ms) activation of Tac1+; CGRP- neurons (top), and quantified changes in breathing frequency over time (bottom; n=6 mice, 50 to100 stimulations per mouse), showing the change in breathing can outlast the duration of the stimulus. Means±SE. Source data and statistical details provided in Source Data file.

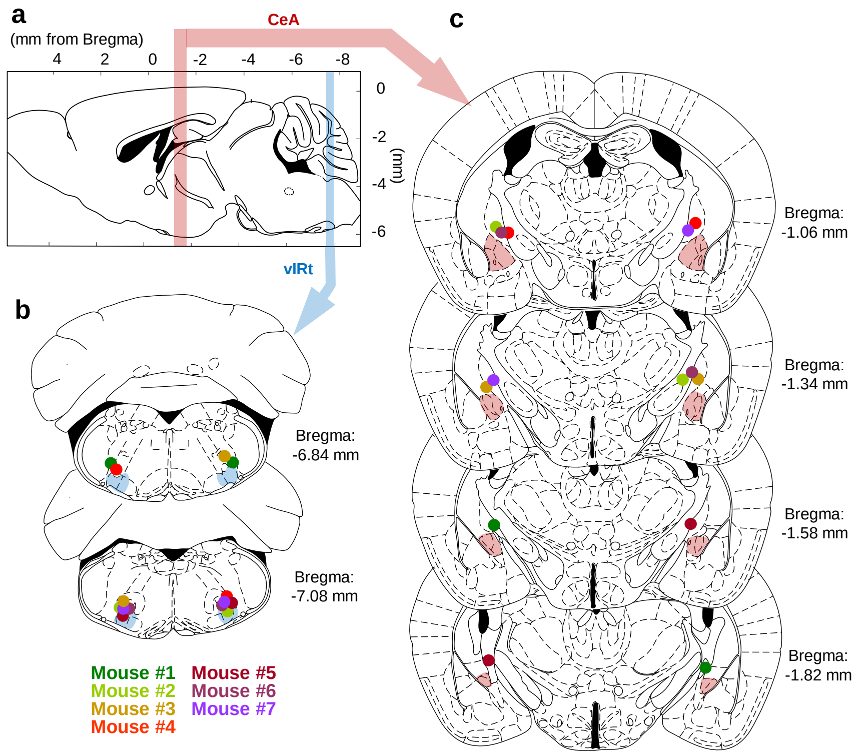

Figure S9: Fiber optic placements for vIRt and CeA projection stimulation experiments. a) Sagittal view of mouse brain showing anterior-posterior Bregma coordinates of the vIRt and CeA. **b** and **c**) Locations of fiber optics for photostimulation of Tac1+; CGRP- PBN neuron terminals in the vIRt (b) and CeA (c). Images adapted from Franklin and Paxinos' "The mouse brain in stereotaxic coordinates" [118].

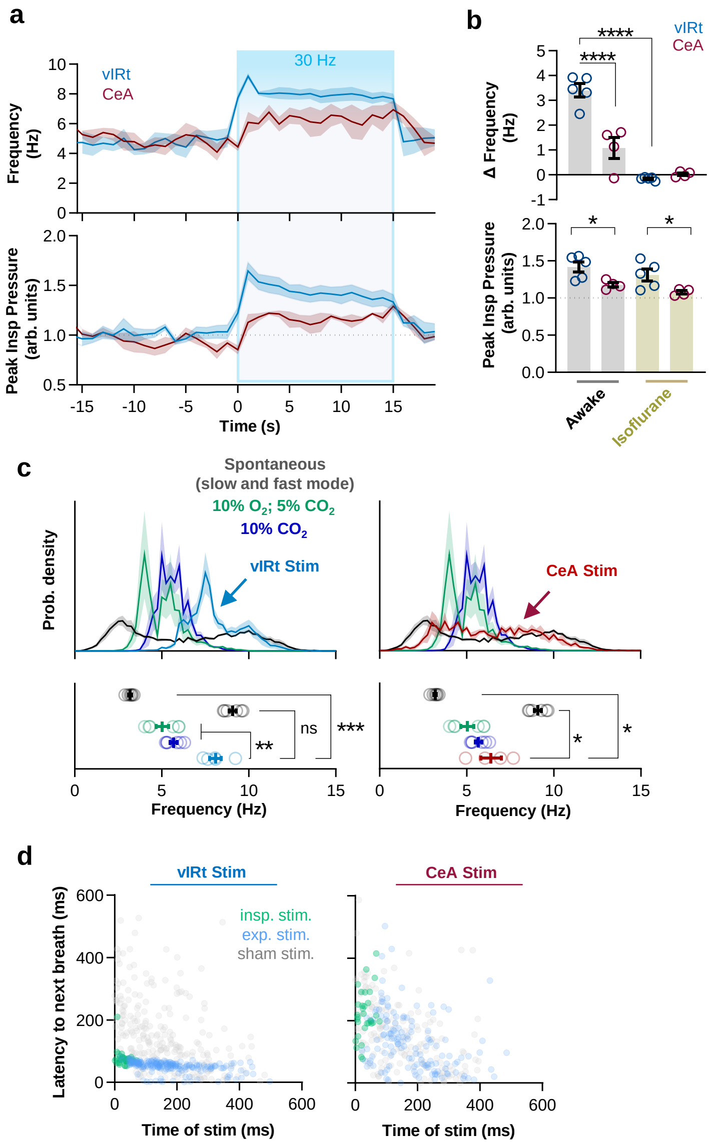

Figure S10: The state-dependent control of breathing by Tac1+;CGRP- PBN neurons is mediated by direct projections to the ventral medulla. **a**) Comparison of breathing frequency (top) and peak inspiratory pressure (bottom; normalized to light OFF condition) during 30-Hz photostimulation of projections of Tac1+; CGRP- PBN neurons to the vIRt (n=5) or CeA (n=4). **b**) Average changes in frequency and peak inspiratory pressure evoked by activation of terminals in the vIRt (n=5 mice) or CeA (n=4 mice) in the awake or anesthetized state. RM two-way ANOVA with Bonferroni's multiple comparisons tests. **c**) Probability density histograms (top) and mean values from each mouse (bottom) comparing breathing frequencies under spontaneous conditions (slow and fast mode) or during near maximal chemoreflex drive (5% CO_2, 10% O_2 or 10% CO_2; n=6 for each condition) with frequencies evoked during stimulation of projections from Tac1+; CGRP- PBN neurons to the vIRt (n=5;left) or the CeA (n=4; right). **d**) Latencies to breath onset following brief 25-ms photostimulation of Tac1+; CGRP- terminals in the vIRt (n=5; left) or CeA (n=4; right) vs. the time of the stimulation relative to the onset of the preceding breath. * p<0.05; ** p<0.01; *** p<0.001; **** p<0.0001. Means±SE. Source data and statistical details provided in Source Data file.

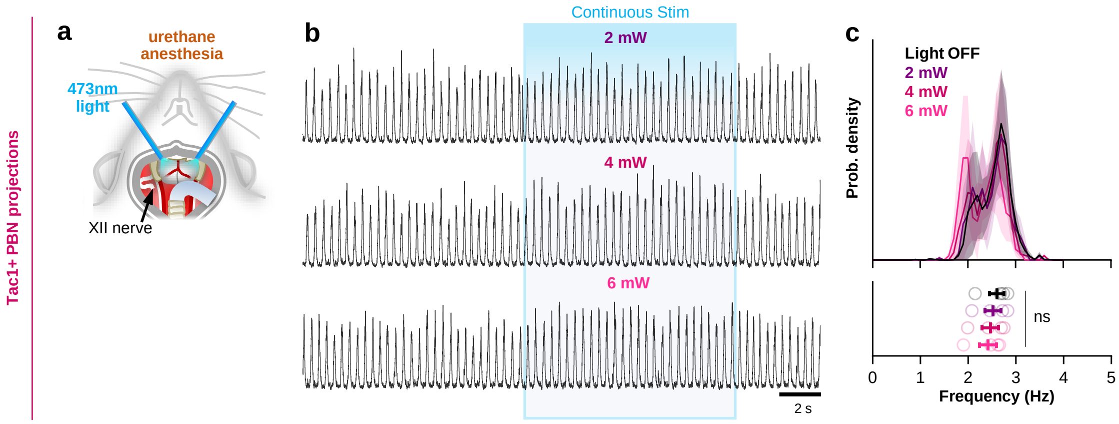

Figure S11: Laser power "dose response" of Tac1 projections in the vIRt. a) Illustration of surgical preparation in a urethane anesthetized mouse. **b**) Example XII nerve recordings during bilateral 10-s continuous stimulation of projections from Tac1+ PBN neurons in the vIRt at 2 mW (similar to power used in awake mice), 4 mW, and 6 mW laser powers (n=4 each condition). **c**) Probability density histogram (top) and mean values from each mouse (bottom; n=4) comparing breathing frequencies during Light OFF and 2-, 4-, and 6-mW stimulations. One-way RM ANOVA with Bonferroni's multiple comparisons tests. ns = not significant. Means±SE. Source data and statistical details provided in Source Data file.

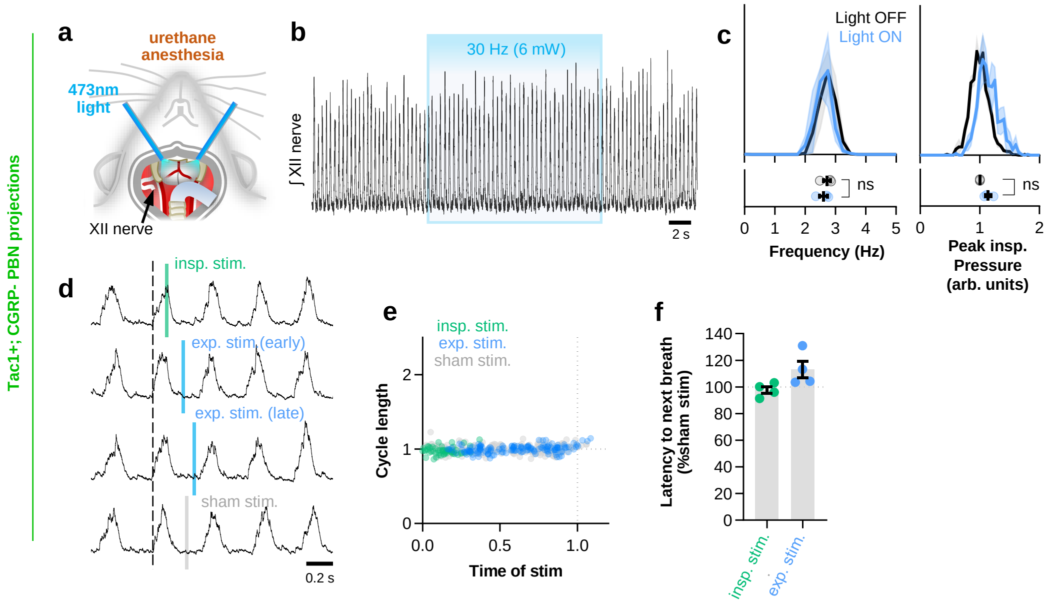

Figure S12: Strong activation of Tac1+; CGRP- terminals in the ventral medulla does not alter breathing in urethane anesthetized mice. **a**) Illustration of surgical approach for access the ventral medullary surface and recording of respiratory motor output from the XII nerve. **b**) Example XII nerve recording showing inspiratory activity (upward deflections) before and during a 15 s 30-Hz photostimulation of Tac1+; CGRP- terminals in the vIRt. Photostimulations were bilateral with 200-µm diameter fiber optics and laser power was set to 6 mW at each fiber tip (n=4). **c**) Probability density histograms (top) and mean values from each mouse (bottom; n=4) comparing breathing frequencies (left) and peak inspiratory pressures (right) during light OFF and light ON conditions. Paired two-tailed t-tests. **d**) Example inspiratory rhythm recorded from the XII nerve during brief (25 ms, 6 mW) light pulses occurring at varied time point of the respiratory cycle. **e**) Plots quantifying the effects of brief photoactivations of Tac1+; CGRP- projections from the PBN to the vIRt on respiratory cycle length relative to the time that the stimulation occurred (abscissa and ordinate both normalized to mean sham respiratory cycle length). Individual stimulations (n=198 from n=4 mice) during inspiration or expiration are represented by green and blue dots, respectively; sham stimulations are show in light gray. **f**) Quantified latency from the time of stimulation to the onset of the next breath expressed as a % of sham stimulations during inspiration (green) or expiration (blue). Two-tailed Wilcoxon matched pairs rank test, n=4 each). ns = not significant. Means±SE. Source data and statistical details provided in Source Data file.
