# Supplementary material for: Parabrachial tachykinin1-expressing neurons involved in state-dependent breathing control
Source: Nat Commun. 2023 Feb 21;14:963. doi: 10.1038/s41467-023-36603-z (PMC9944916; doi:10.1038/s41467-023-36603-z)
Supplement: Supplementary file 3 — Reporting Summary [file 41467_2023_36603_MOESM3_ESM.pdf]

nature portfolio

Corresponding author(s): Nathan Baertsch

Last updated by author(s): Jan 26, 2023

Reporting Summary

Nature Portfolio wishes to improve the reproducibility of the work that we publish. This form provides structure for consistency and transparency in reporting. For further information on Nature Portfolio policies, see our Editorial Policies and the Editorial Policy Checklist.

Statistics

For all statistical analyses, confirm that the following items are present in the figure legend, table legend, main text, or Methods section.

n/a	Confirmed	
☐	☒	The exact sample size (*n*) for each experimental group/condition, given as a discrete number and unit of measurement
☐	☒	A statement on whether measurements were taken from distinct samples or whether the same sample was measured repeatedly
☐	☒	The statistical test(s) used AND whether they are one- or two-sided<br>*Only common tests should be described solely by name; describe more complex techniques in the Methods section.*
☒	☐	A description of all covariates tested
☐	☒	A description of any assumptions or corrections, such as tests of normality and adjustment for multiple comparisons
☐	☒	A full description of the statistical parameters including central tendency (e.g. means) or other basic estimates (e.g. regression coefficient) AND variation (e.g. standard deviation) or associated estimates of uncertainty (e.g. confidence intervals)
☐	☒	For null hypothesis testing, the test statistic (e.g. *F*, *t*, *r*) with confidence intervals, effect sizes, degrees of freedom and *P* value noted<br>*Give P values as exact values whenever suitable.*
☒	☐	For Bayesian analysis, information on the choice of priors and Markov chain Monte Carlo settings
☒	☐	For hierarchical and complex designs, identification of the appropriate level for tests and full reporting of outcomes
☒	☐	Estimates of effect sizes (e.g. Cohen's *d*, Pearson's *r*), indicating how they were calculated

Our web collection on statistics for biologists contains articles on many of the points above.

Software and code

Policy information about availability of computer code

Data collection	All software used for data collection is described in Methods. pClamp 9 (molecular devices)
Data analysis	All commercial software used is described in Methods. GraphPad Prism 9, Microsoft Excel version 2212, MatLab R2022a, R Studio v2021.09, CellProfiler 4.2.5

For manuscripts utilizing custom algorithms or software that are central to the research but not yet described in published literature, software must be made available to editors and reviewers. We strongly encourage code deposition in a community repository (e.g. GitHub). See the Nature Portfolio guidelines for submitting code & software for further information.

Data

Policy information about availability of data

All manuscripts must include a data availability statement. This statement should provide the following information, where applicable:

- Accession codes, unique identifiers, or web links for publicly available datasets
- A description of any restrictions on data availability
- For clinical datasets or third party data, please ensure that the statement adheres to our policy

All data is included within the manuscript and associated source data files.

Human research participants

Policy information about studies involving human research participants and Sex and Gender in Research.

Reporting on sex and gender	n/a
Population characteristics	n/a
Recruitment	n/a
Ethics oversight	n/a

Note that full information on the approval of the study protocol must also be provided in the manuscript.

Field-specific reporting

Please select the one below that is the best fit for your research. If you are not sure, read the appropriate sections before making your selection.

☒ Life sciences ☐ Behavioural & social sciences ☐ Ecological, evolutionary & environmental sciences

For a reference copy of the document with all sections, see nature.com/documents/nr-reporting-summary-flat.pdf

Life sciences study design

All studies must disclose on these points even when the disclosure is negative.

Sample size	Sample sizes were chosen based on power analysis and variance from previous studies using similar methods (e.g. Bowen el al., 2020; Olivera et al., 2021). Based on the magnitude and consistency of the effects observed in pilot experiments for this study, we expected n=5 per group would provide sufficient statistical power. However, group sizes were sometimes larger if the litters produced for a given experiment contained more than 5 pups.
Data exclusions	no data were excluded from analysis
Replication	all experiments were replicated at least three times
Randomization	male and female mice were allocated randomly into experimental groups when applicable
Blinding	Investigators were not blinded during data collection or analysis. Mice require monitoring by the experimenter during stimulations and the stimulations produce very obvious effects on breathing making it impossible to blind the researchers during data collection or analysis.

Reporting for specific materials, systems and methods

We require information from authors about some types of materials, experimental systems and methods used in many studies. Here, indicate whether each material, system or method listed is relevant to your study. If you are not sure if a list item applies to your research, read the appropriate section before selecting a response.

Materials & experimental systems

n/a	Involved in the study	
☐	☒	Antibodies
☐	☒	Eukaryotic cell lines
☒	☐	Palaeontology and archaeology
☐	☒	Animals and other organisms
☒	☐	Clinical data
☒	☐	Dual use research of concern

Methods

n/a	Involved in the study	
☒	☐	ChIP-seq
☒	☐	Flow cytometry
☒	☐	MRI-based neuroimaging

Antibodies

Antibodies used	chicken-anti-GFP (1:10,000, Abcam, Cat #: ab13970), rabbit-anti-DsRed (1:1000, TaKaRa, Cat #: 632496), Alexa Fluor 488 donkey anti-chicken (1:1000,Jackson ImmunoResearch Cat# 703-545-155) and Alexa Fluor 594 donkey anti-rabbit (1:1000 Jackson ImmunoResearch, Cat# 711-585-152).

Validation	Both primary antibodies were used to enhance signal from genetically encoded florescent proteins and therefore signal patterns before and after antibody stain were used for validation. Chicken polyclonal anti-GFP validated by manufacturer for wholemount IHC of mouse tissue. Rabbit monoclonal anti-dsRed first validated in mice by Tseng et al., 2010 (PMID 20575070) and cited in over 225 peer reviewed articles since.

Eukaryotic cell lines

Policy information about cell lines and Sex and Gender in Research

Cell line source(s)	HEK 293 cells were sourced from cell bank originally sourced from ATCC.
Authentication	HEK 293 cells were not authenticated.
Mycoplasma contamination	HEK 293 cells tested negative for mycoplasma contamination.
Commonly misidentified lines (See ICLAC register)	*Name any commonly misidentified cell lines used in the study and provide a rationale for their use.*

Animals and other research organisms

Policy information about studies involving animals; ARRIVE guidelines recommended for reporting animal research, and Sex and Gender in Research

Laboratory animals	Experiments were conducted on adult (P78-P135) male and female C57Bl/6J mice. Tac1-Cre, Calca-Cre, Oprm1-Cre, and Calca-FLPo transgenic lines were used. Mice were bred and housed in a temperature (65-75F) and humidity (40-60%) controlled vivarium on a 12-hr light cycle with access to food and water ad libitum.
Wild animals	No wild animals used.
Reporting on sex	Male and female mice were both used and chosen at random. Optogenetic manipulations caused dramatic effects on breathing in both sexes with no sex-dependent trends noticeable. Formal analysis was not performed to determine if any subtle sex-dependent differences exist in the data due to limited sample size.
Field-collected samples	No field collected samples used.
Ethics oversight	University of Washington IACUC and Seattle Children's Research Institute IACUC. Experiments done in accordance with the with the Guide for The Care and Use of Animals in Research.

Note that full information on the approval of the study protocol must also be provided in the manuscript.
